# Breast cancer brain metastases: new directions in systemic therapy

**DOI:** 10.3332/ecancer.2013.307

**Published:** 2013-04-18

**Authors:** Nancy U Lin

**Affiliations:** Dana-Farber Cancer Institute, Boston, MA, USA

**Keywords:** brain and nervous system, breast

## Abstract

The management of patients with brain metastases from breast cancer continues to be a major clinical challenge. The standard initial therapeutic approach depends upon the size, location, and number of metastatic lesions and includes consideration of surgical resection, whole-brain radiotherapy, and stereotactic radiosurgery. As systemic therapies for control of extracranial disease improve, patients are surviving long enough to experience subsequent progression events in the brain. Therefore, there is an increasing need to identify both more effective initial treatments as well as to develop multiple lines of salvage treatments for patients with breast cancer brain metastases. This review summarises the clinical experience to date with respect to cytotoxic and targeted systemic therapies for the treatment of brain metastases, highlights ongoing and planned trials of novel approaches and identifies potential targets for future investigation.

## Introduction

Historically, brain metastases were associated with very poor survival [1]. While brain metastases remain incurable, there is increasing optimism that advances in local and systemic therapies may provide clinical benefit in some subsets of patients, and there is increasing interest in exploring novel approaches to the prevention and management of this challenging clinical problem. This review summarises the clinical experience to date with respect to cytotoxic and targeted systemic therapies, highlights ongoing and planned trials and discusses potential targets and novel trial designs for future investigation.

## Incidence and prognosis

Brain metastases are diagnosed in approximately 15% of unselected patients with advanced breast cancer [2]. Over time, it has become increasingly clear that the biology of the primary tumour influences the pattern of metastatic spread, including the likelihood of relapse in the central nervous system (CNS) [3–5]. As many as half of patients with HER2-positive advanced breast cancer will develop brain metastases at some point in the course of their disease [6–8]. Within the HER2-positive subset, hormone receptor status appears to further define the risk of CNS relapse, with patients having hormone receptor-negative/HER2-positive tumours experiencing increased risk of the CNS as site of first relapse compared with patients with hormone receptor-positive/HER2-positive tumours [9]. Patients with metastatic, triple-negative (ER, PR and HER2 negative) breast cancer are also at high risk, with 25–46% of patients developing brain metastases at some point in the course of their disease [4, 8]. Of interest, the timing of the CNS relapse also appears to vary by tumour subtype. Patients with non-luminal tumours (e.g. triple-negative cancers) appear to experience a shorter time to CNS relapse compared with patients with luminal tumours [5, 10].

In historical series, the median survival of unselected patients with breast cancer brain metastases treated with whole-brain radiotherapy (WBRT) has been reported at approximately five to six months [11]. More recent analyses have identified performance status and biologic subtype as major drivers of prognosis. For example, in a multi-institutional retrospective database of over 400 patients with breast cancer brain metastases, a prognostic model (the Diagnosis-Specific Graded Prognostic Assessment, DSGPA) using these factors (plus age) was able to distinguish between patients experiencing a two year median survival versus those with 3.4 months median survival [12, 13]. Across multiple retrospective studies, the most striking differences have been consistently noted between patients with HER2-positive breast cancer (who carry the most favourable prognosis) and those patients with triple-negative breast cancer [14–19]. Based on several lines of evidence, it is likely that improved systemic tumour control is a major contributing factor to this difference. First, although one must interpret retrospective data cautiously because of issues with patient selection, it has been observed by multiple groups that patients with HER2-positive tumours who continue on anti-HER2 therapy following the diagnosis of brain metastases fare better than those who received either no therapy, or chemotherapy without HER2-directed therapy [18, 19]. Second, as many as half of patients with HER2-positive brain metastases suffer a death primarily related to CNS (as opposed to systemic) progression; this is distinctly different from patients with triple-negative brain metastases, where patients most commonly die of uncontrolled systemic disease [6, 17].

## Current standard of care

A full discussion of the current standard of care approaches is outside the scope of this review. Treatment guidelines for patients with brain metastases from solid tumours are available through the National Comprehensive Cancer Network (NCCN) and professional societies [20, 21]. Because of the relative paucity of breast cancer-specific prospective trials in this patient population, the recommendations are largely based upon data generated from trials of patients with solid tumours (of which lung cancer is most highly represented).

In general, the initial management of patients with brain metastases depends on (a) the number, size, and location of brain lesions; (b) the presence or absence of neurological symptoms; (c) the patient’s performance status and medical comorbidities; (d) the status of systemic metastases; (e) the availability of systemic treatment options; and (f) patient preference. In general, initial management will include some combination of surgical resection, radiosurgery, and/or WBRT, depending on the above factors. Systemic therapy could be a consideration either on a clinical trial, in the context of minimal CNS disease burden with rapidly progressive extracranial disease, or in select, well-informed patients as an alternative to localised therapies with close follow-up (i.e. a patient with small, asymptomatic CNS lesions). Among patients who have developed subsequent CNS progression after initial standard therapy, options include surgical resection, WBRT, stereotactic radiosurgery (SRS), off-label use of systemic therapy, consideration of a clinical trial, or best supportive care. Options will vary based on prior treatments received, response to prior treatments, location and number of the new or progressive CNS lesions, and the other patient and disease-related factors as listed above. For both initial and subsequent management decisions, a multidisciplinary approach is essential in making recommendations based upon an assessment of the patient in the context of their local therapy options, extracranial disease status, planned or ongoing systemic therapy, and clinical trial options.

## Available data for systemic chemotherapy approaches

At present, there are no FDA-approved systemic therapies for the treatment of breast cancer brain metastases. A number of small, prospective trials of cytotoxic chemotherapy have been completed and are summarised in Table 1.

Temozolomide has been examined in a phase II trial of the National Cancer Institute of Canada Clinical Trials Group (NCI-CTG) [22]. This trial included 19 women with heavily pretreated metastatic breast cancer (MBC) and allowed patients with brain metastases (*n*=5) to participate. No objective responses were observed and the study was closed to further accrual due to lack of sufficient activity. A dose-dense temozolomide regimen has also been evaluated in a phase II study of solid tumour patients (*n*=51 with breast cancer) [23]. The objective response rate among breast cancer patients was 4%; however, the majority of the responses were transient, and median progression-free survival (PFS) was approximately two months.

Capecitabine is an active agent in patients with MBC. With respect to breast cancer brain metastases, capecitabine has been evaluated in a phase I study in combination with temozolomide, with a reported response rate of 18% [24]. CNS activity has also been reported in a case series from Memorial Sloan-Kettering Cancer Center, with capecitabine given as a single agent [25].

Platinum agents have also been prospectively evaluated, although in combination with other cytotoxic agents rather than as monotherapy. In a phase II study evaluating the combination of cisplatin and temozolomide, six out of 15 (40%) of breast cancer patients achieved a partial CNS response [26]. For the 32 total patients enrolled in the study, median time to progression (TTP) was 2.9 months. In a separate study evaluating the combination of cisplatin and etoposide, the CNS response rate was 38% with a median TTP of four months [27]. It should be noted that in the latter study, patients were not allowed to have received prior CNS radiotherapy, and about one-third were chemotherapy naive. Additionally, although there has been increasing interest in the use of platinum agents in certain subsets of breast cancer, the systemic response rate in unselected, pretreated patients has been low [28]. Still, for patients who have seen relatively little prior systemic therapy and who have progressive brain metastases, off-label use of platinum agents could be a plausible treatment option.

Epothilones are tubulin-stabilising agents with activity against multiple solid tumours. At present, only ixabepilone is commercially available and is approved for the treatment of refractory MBC. Negative results of a trial in patients with primary brain tumours and the existence of investigation alepothilones with excellent blood–brain barrier (BBB) permeability have limited efforts to formally test the effects of ixabepilone in patients with breast cancer brain metastases [29]. Sagopilone is an epothilone B analogue that readily crosses the BBB and is not a P-glycoprotein substrate [30]. Despite its preclinical promise, its single-agent activity in the phase II study breast cancer brain metastasis study was only modest, and there currently are no plans to further develop the drug in this setting [31]. Patupilone, another BBB-permeable epothilone, has also demonstrated CNS activity in breast cancer, and future trials are under consideration [32].

Finally, some regimens, while not evaluated in prospective clinical trials, have reported efficacy in case series and include CMF (cyclophosphamide, methotrexate and 5-fluorouracil), anthracycline-based regimens and high-dose intravenous methotrexate [33, 34].

## Available data for targeted therapy approaches

Endocrine therapies remain the oldest and most established targeted treatments for the management of patients with breast cancer. In the setting of CNS disease, there have been multiple case reports describing responses to tamoxifen, aromatase inhibitors and megestrol acetate, although no prospective trials have been conducted [35–37]. Unfortunately, the reality is that most patients with ER-positive breast cancers have developed endocrine-refractory disease by the time brain metastases are detected. However, among the subset who have had minimal prior endocrine therapy exposure and/or who have had prior sustained responses to treatment, endocrine therapy may be an option that could be considered.

Trastuzumab does not appear to cross the intact BBB [38]. Although it should be acknowledged that trastuzumab levels have not been directly measured in human brain metastasis samples, nevertheless, clinically, it has been repeatedly observed that a substantial proportion of patients with HER2-positive MBC develop isolated CNS progression in the setting of continued systemic disease control on trastuzumab [6]. A number of studies have evaluated the efficacy of lapatinib, a small molecule tyrosine kinase inhibitor that targets both EGFR and HER2, in the setting of HER2-positive breast cancer brain metastases (Table 2). The response rate to single-agent lapatinib in the refractory setting was underwhelming; however, when given in combination with capecitabine, CNS response rates ranging from 18% to 38% have been reported [39–44]. More recently, lapatinib plus capecitabine has been studied in the upfront setting in the LANDSCAPE trial, prior to the use of WBRT or other local therapies for the treatment of brain metastases. Approximately two-thirds of patients achieved a CNS objective response, and the median TTP was 5.5 months, with one-year survival exceeding 70% (Bachelot *et al*, Lancet Oncology, in press) [45]. Results of this study have prompted ongoing European efforts to organise a potential phase III trial directly comparing lapatinib plus capecitabine with WBRT for initial treatment of patients with HER2-positive brain metastases, a study which will be needed before this regimen becomes part of routine upfront care.

Lapatinib plus capecitabine has also been compared with trastuzumab plus capecitabine in the CEREBEL trial, with a primary endpoint of the development of CNS metastases [46]. Of note, patients were required to have CNS screening at baseline, and this led to 20% of the study patients having asymptomatic brain metastases detected, leading to study exclusion. The study did not meet its primary endpoint because of a paucity of CNS events in both arms. It is likely that the study was inconclusive as the highest-risk population for developing brain metastases was screened out due to the screening procedures.

## New targets and avenues of investigation

Despite some notable advances in recent years, there remains much progress to be made. Commercially available treatment options for patients with progressive brain metastases after surgical or radiotherapy approaches remain limited, and there are still no systemic regimens that have gained a formal indication in this setting. Fortunately, the landscape is changing. There are a number of novel agents of interest and an increasing number of trials designed to evaluate the efficacy of these compounds in the CNS (Table 3).

The ideal agent would reach therapeutic concentrations in the brain, be active against breast cancer in both intracranial and extracranial sites (including in patients who have received multiple prior lines of systemic therapy), have a favourable toxicity profile, and demonstrably benefit patients. Two examples are the third-generation taxane TPI-287 and the peptide-taxane conjugate GRN1005. TPI-287 is a microtubule-stabilising agent designed to circumvent the MDR-1 drug efflux resistance mechanism and is also highly permeable across the intact BBB. In a preclinical model using intracardiac injection of 231-BR cells, treatment with TPI-287 inhibited outgrowth of brain metastases, an effect that was not seen with paclitaxel, ixabepilone, or nab-paclitaxel [47]. GRN1005 (previously ANG1005) is a peptide (angiopep-2)-taxane conjugate that targets the lipoprotein receptor-related protein 1, which is upregulated on the surface of the BBB as well as in brain metastases, and reaches therapeutic concentrations in the brain in preclinical models [48, 49]. In the phase I study in patients with brain metastases from solid tumours, CNS activity was observed across multiple tumour types, including breast cancer and patients with taxane-refractory disease [50]. The most commonly observed adverse events were neutropenia, anaemia, and fatigue. Based upon the data outlined above, phase II trials of both TPI-287 and GRN1005 are now ongoing. However, interim analysis of the phase II trial of GRN 1005 in breast cancer brain metastases did not meet its efficacy endpoint. Follow up and analysis of the entire study population will be required before reaching conclusions about the future of the compound.

In light of the activity observed with lapatinib, other HER2-directed tyrosine kinase inhibitors are also being evaluated for CNS activity. For example, neratinib has been evaluated in phase II trials in HER2-positive MBC patients for the treatment of extracranial disease. Approximately half of patients treated in the first-line setting achieved an objective response; in the refractory setting, the response rate was 24%, with a median PFS of 22 weeks [51]. Based on these promising findings, neratinib is now being evaluated in a multicentre study for the treatment of patients with HER2-positive breast cancer and progressive CNS metastases. Similarly, afatinib is an irreversible inhibitor of EGFR and HER2, with preliminary evidence of activity in the phase II non-CNS metastatic setting, which is currently in clinical trials for the treatment of breast cancer brain metastases [52]. Other HER2-targeting compounds of interest, including those with excellent BBB penetration, are in the early stages of clinical development.

Beyond HER2, other targets of interest include tumour-associated angiogenesis, the PI3K/mTOR pathway, and the DNA repair pathway. With respect to angiogenesis, it is well documented that breast cancer brain metastases are highly angiogenic and associated with abnormal tumour-associated vasculature [53]. In preclinical models, VEGF promotes the growth of breast cancer brain metastases, and this growth is inhibited by anti-angiogenic agents [54]. There are preliminary data supporting an effect of bevacizumab on brain metastases and reassuring safety data from the standpoint of CNS haemorrhage [55, 56]. Moreover, bevacizumab has an established role in the treatment of primary brain tumours [57, 58]. It is expected that additional data regarding the efficacy of bevacizumab/chemotherapy combinations for the treatment of breast cancer brain metastases will be available within the next year (NCT01004172; NCT01281696) and, if promising, could lead to additional trials in the near future. It should be noted that radiographic response to bevacizumab should be interpreted with some caution, given its anti-oedema effects, which may lead to decreases in gadolinium leakage and potential under-calling of tumour burden. While in patients with primary brain tumours, specific modifications of response criteria have been added to account for some of these issues, there are as of yet no standard criteria for assessment of CNS response in the setting of solid tumour brain metastases [59].

Another pathway of considerable active interest in breast cancer is the PI3K/mTOR pathway. Earlier this year, everolimus, a rapamycin analogue that inhibits mTOR signalling, was approved in combination with exemestane for patients with ER-positive MBC, on the basis of a randomised trial demonstrating a substantial improvement in PFS [60]. In a randomised phase II trial, everolimus also demonstrated additive benefit when given in conjunction with tamoxifen [61]. Everolimus is also under active investigation [62, 63] in the setting of HER2-positive breast cancer. Notably, everolimus has demonstrated efficacy against a rare type of brain tumour (subependymal giant cell astrocytoma, SEGA), suggesting it reaches therapeutic levels in the brain [64]. A phase II study evaluating everolimus (in combination with trastuzumab and vinorelbine) among patients with HER2-positive breast cancer brain metastases is ongoing.

In tandem with ongoing studies evaluating mTOR inhibitors in breast cancer, there is a tremendous amount of effort being placed in evaluating pan- and alpha-specific PI3 kinase inhibitors. Approximately one-third of HER2-positive breast cancers contain a PIK3CA somatic mutation, and PTEN loss is present in up to half of triple-negative breast cancers [65]. PIK3CA mutations are also commonly present in ER-positive breast cancers [66]. Of interest, anecdotal reports of CNS activity have been reported with BEZ235 and BKM120 in a patient with HER2-positive and triple-negative breast cancer, respectively [67, 68]. In particular, BKM120 is associated with mood alterations that are thought to be related its ability to cross the intact BBB (Zhao J, personal communication, September 2012), and there is interest in further evaluating this compound in the CNS.

Finally, although the enthusiasm for PARP inhibitors waned somewhat with the presentation of the negative phase III iniparib trial, it now appears that iniparib is not a true PARP inhibitor [69]. Other PARP inhibitors have shown clear single-agent activity in BRCA mutation carriers, which is notable given reports of a high incidence of brain metastases in BRCA carriers with breast cancer [70–72]. Their role in non-BRCA-associated breast cancer is still uncertain, but it is an area of continued active investigation. A phase I study of the PARP inhibitor ABT-888 in combination with WBRT is ongoing, and there is interest in evaluating the combination of PARP inhibitors plus chemotherapy or other targeted approaches for brain metastases in the future.

## Leptomeningeal metastases

Among patients with leptomeningeal metastases, breast cancer is the most commonly represented solid tumour [73]. Still, leptomeningeal metastases are a fairly infrequent occurrence in most breast cancer patients. In series from Korea and the United States, only 3–5% of patients with HER2-positive MBC were reported to have developed leptomeningeal metastases [6, 7, 74]. The incidence does appear higher than this in patients with ER-positive lobular breast cancers and in patients with triple-negative breast cancer, although the point estimates have varied [4, 75–77].

There are very few prospective trials to guide therapy. Unfortunately, survival remains poor across breast cancer subtypes [78]. Intrathecal chemotherapy can be effective, but even with good patient selection, long-term responses are uncommon [79, 80]. Case reports and case series have been published on the experience with endocrine therapy, high-dose intravenous methotrexate, capecitabine, and irinotecan, and off-label use could be considered in select circumstances [25, 81–83]. Finally, in patients with elevated intracranial pressure, placement of a ventriculoperitoneal shunt can have clear palliative benefits and is a consideration within the context of multidisciplinary management [84, 85].

From an investigational standpoint, given that HER2 amplification appears to be retained in patients with HER2-positive primary tumours and leptomeningeal involvement, intrathecal trastuzumab is the subject of two ongoing prospective trials in France and in the United States (www.clinicaltrials.gov; NCT01373710, NCT01325207) [86]. A number of case reports have been published in the medical literature, although on close review, in many cases, patients received multiple concurrent therapies, making isolation of the true effect of intrathecal trastuzumab a challenge [87–89]. At present, off-label use of intrathecal trastuzumab is not recommended, given that the commercial drug supply is not formulated for intrathecal use [90]. Newer anti-HER2 agents, such as pertuzumab or TDM1, which have demonstrated activity against extracranial metastases, could also be of interest in patients with leptomeningeal disease when given intrathecally. However, these would need to be studied carefully for both safety and efficacy and, as of now, would not be recommended for off-label use in this setting [91–93].

## Challenges and opportunities in clinical trial design

Patients with brain metastases have historically been excluded from the vast majority of clinical trials in cancer. As patients with brain metastases are included in trials and as more trials are opened that specifically assess the CNS efficacy of novel treatments, there is an increasing need to standardise response criteria for assessment of brain metastases. Because neither the RECIST nor Macdonald criteria were developed for the specific purpose of evaluating patients with CNS metastases, there are some gaps and inconsistencies in how they are applied in brain metastasis studies, and many groups have either modified the criteria or developed new criteria. The Response Assessment in Neuro-Oncology (RANO) Metastatic Working Group is currently taking on the task of formulating new response guidelines in an effort to reduce this heterogeneity across studies.

A traditional clinical trial paradigm has been to evaluate novel systemic approaches in the refractory setting (i.e., in patients whose CNS disease has progressed after radiotherapy) in patients with measurable disease and with objective response as the primary endpoint. Provocative data in a small study of patients treated with lapatinib and capecitabine as upfront therapy for HER2-positive brain metastases raise the question of whether systemic therapy might someday take the place of radiotherapy-based approaches and raise difficult questions as to what type of data would be required to comfortably make that shift [45]. In particular, in a hypothetical phase III study directly comparing WBRT with systemic therapy, CNS response, or CNS progression-free survival may not be the most appropriate primary endpoint(s). In a small, randomised study comparing SRS alone to SRS with WBRT, although intracranial control was superior in the SRS + WBRT arm, neurocognitive outcomes were inferior, suggesting that intracranial control is not directly translatable to improved patient outcomes [94]. Yet, designating progression-free survival as the primary endpoint leaves open questions about the allowable systemic therapies in the WBRT arm and about the treatment of CNS and non-CNS progression in the statistical plan.

Another trial design that has been proposed is to consider ‘secondary prevention’; that is, to assess the ability of novel approaches to prevent further CNS progression among patients with a limited number of brain metastases treated with SRS alone. To date, there have not been any clinical breast cancer studies utilising this model, although there is significant interest in launching such a study. Ultimately, it would be ideal to develop therapies that could prevent the occurrence of brain metastases altogether. For example, in preclinical models, targeting of polo-like kinase 1, α(V)-integrins, b-raf, and histone deacetylase decreases brain metastatic outgrowth and could be potentially directed translated into clinical trials in the future [95–98].

## Conclusions

Breast cancer brain metastases continue to pose a difficult challenge for patients and clinicians. For the majority of patients, the most appropriate initial therapy remains a radiotherapy-based approach, although select patients may benefit from surgery, and systemic therapy could be considered in some circumstances. Although there are still no systemic therapies with an FDA indication for the treatment of breast cancer brain metastases, there is emerging evidence of activity of a number of regimens, and there are an increasing number of clinical trials targeting this patient population.

## Figures and Tables

**Table 1:** Prospective trials of chemotherapy for breast cancer brain metastases.

Regimen	Number of Patients (Number of Breast Cancer Patients)	Patient Population	CNS ORR in Breast Cancer Subset	TTP/PFS
Temozolomide [22]	19 (5)	Pretreated with systemic therapy	0%	<2 months
Temozolomide [23]	157 (51)	80% prior chemotherapy for MBC 24% prior WBRT	4%	~2 months
Capecitabine + temozolomide [24]	24 (24)	33% prior WBRT	18%	3 months
Cisplatin + temozolomide [26]	32 (15)	~50% prior WBRT	40%	2.9 months
Cisplatin + etoposide [27]	107 (56)	No prior CNS RT allowed; 36% chemotherapy naive	38%	4 months
Sagopilone [31]	15 (15)	Progression after CNS RT required	13%	1.4 months
Patupilone [32]	36 (36)	Progression after CNS RT required	19%	2.8 months
Vinorelbine + temozolomide [99]	38 (11)	Heavily pretreated patients	0% (1 minor response observed)	1.9 months

a MBC, metastatic breast cancer.

b WBRT, whole-brain radiotherapy.

c c CNS, central nervous system.

d RT, radiotherapy.

**Table 2:** Studies of Lapatinib in HER2-positive breast cancer brain metastases.

Regimen	Number of Patients	Patient Population	CNS ORR	TTP/PFS
Lapatinib [39]	39	Heavily pretreated	2.6%	3.0 months
Lapatinib [40]	237	Progression after CNS RT required	6%	2.4 months
Lapatinib + capecitabine [40]	50	Progression after CNS RT and through lapatinib monotherapy required	20%	3.6 months
Lapatinib + capecitabine [41]	138	Heavily pretreated	18%	NR
Lapatinib + capecitabine [43]	34	Heavily pretreated	21%	5.1 months
Lapatinib + capecitabine [42]	22	Heavily pretreated	32%	5.1 months
Lapatinib + capecitabine [44]	13	Heavily pretreated	38%	NR
Lapatinib + capecitabine [45]	45	No prior CNS radiotherapy allowed	67%	5.5 months
Lapatinib + topotecan [44]	9	Heavily pretreated	0%	NR
Lapatinib + temozolomide [100]	17	Heavily pretreated	NR	2.8 months

NR, not reported; CNS, central nervous system

**Table 3:** Selected trials for breast cancer brain metastases.

Agent	Phase of Trial	Class or Target	Patient Population	ClinicalTrials.gov Identifier
TPI-287	II	Taxane	Breast cancer, all subtypes	NCT01480583
GRN1005	II	Taxane-peptide conjugate	Breast cancer, all subtypes	NCT01480583
2B3-101	I	Glutathione-pegylatedlipsosomal doxorubicin	Solid tumours and malignant glioma	
Neratinib	II	HER2-directed TKI	Breast cancer, HER2-positive	NCT01494662
Afatinib	II	HER2-directed TKI	Breast cancer, HER2-positive	NCT01441596
Bevacizumab + carboplatin	II	VEGF inhibitor	Breast cancer, all subtypes	NCT01004172
Bevacizumab + cisplatin + etoposide	II	VEGF inhibitor	Breast cancer, all subtypes	NCT01281696
Everolimus + trastuzumab + vinorelbine	II	mTOR inhibitor	Breast cancer, HER2-positive	NCT01305941
ABT-888 + WBRT	I	PARP inhibitor	Solid tumours	NCT00649207

NR, not reported; CNS, central nervous system
